# Dengue Virus Serotype 4, Roraima State, Brazil

**DOI:** 10.3201/eid1705.101681

**Published:** 2011-05

**Authors:** José Gomes Temporão, Gerson Oliveira Penna, Eduardo Hage Carmo, Giovanini Evelim Coelho, Raimunda do Socorro Silva Azevedo, Márcio Roberto Teixeira Nunes, Pedro Fernando da Costa Vasconcelos

**Affiliations:** Author affiliations**:** Ministry of Health Brasília, Brasilia, Brazil (J.G. Temporão, G.O. Penna);; University of Brasília, Brasilia (G.O. Penna);; Secretariat of Health Surveillance, Brasilia (E.H. Carmo, G.E. Coelho);; Evandro Chagas Institute, Ananindeua, Brazil (R. do Socorro Silva Azevedo, M.R.T. Nunes, P.F. da Costa Vasconcelos)

**Keywords:** dengue 4, virus isolation, genetic characterization, genotype II, viruses, letter

**To the Editor:** In July 2010, dengue virus serotype 4 (DENV-4) reemerged in Boa Vista, the capital of Roraima State, in northern Brazil (Figure, panel A), after an absence of 28 years (1). Cases were identified during late June in the municipalities of Boa Vista and Cantá. For all patients, the clinical course of disease was classic, and all recovered uneventfully. The most commonly reported signs and symptoms were fever, headache, chills, muscle and joint pains, rash, nausea and vomiting, and retro-ocular pain. Patient ages were 11–51 years (median 31 years); 5 patients were male.

**Figure:**
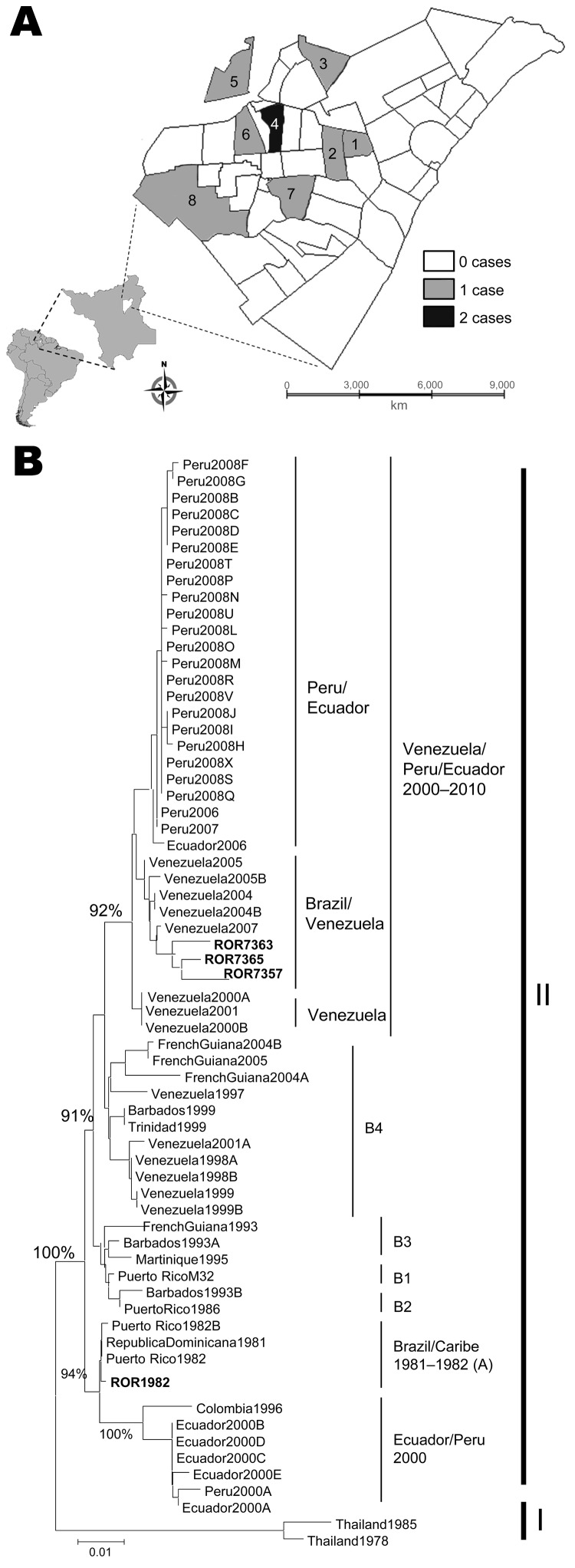
A) Boa Vista, Roraima State, Brazil, showing the districts where dengue virus type 4 (DENV-4) was isolated. 1, Liberdade; 2, Buritis; 3, Cauamé; 4, Santa Tereza; 5, Cidade Satélite; 6, Dr. Silvio Leite; 7, Joquel Clube; 8, Sen. Hélio Campos. B) Phylogenetic analysis of the DENV-4 envelope gene sequences (in **boldface**) constructed by using the neighbor-joining method, showing the cluster formed by Boa Vista and Venenzuela DENV-4 genotype I strains. Bootstrap values were set for 1,000 replicates and are placed over each main node of the tree. ROR1982 represents the DENV-4 isolated in Roraima State during the 1982 epidemic. A to B1–B4 represent genotype I subclades. Thailand 1978 and Thailand 1985 strains represent the Asian genotype II. Scale bar corresponds to 1% nucleotide sequence divergence.

Because of the clinical picture, dengue was suspected, and serum samples were collected and sent to Instituto Evandro Chagas for virus isolation. Of 10 DENV strains recovered, 9 were isolated into C6/36 cell samples as described elsewhere (2). Cytopathic effects were observed on days 5–7 postinfection, and virus isolation was confirmed by use of indirect immunofluorescent assay (3). Molecular approaches led to diagnosis of 10 cases. Viral RNA was recovered from infected cells in the supernatant by using a QIAquick viral RNA extraction kit (QIAGEN, Valencia, CA, USA); genome amplification was performed by using a 1-step reverse transcription–PCR (RT-PCR) (4) and 2 set of oligonucleotides designed to amplify the entire N gene in overlapping PCR products. PCR amplification was performed on 5 DENV strains, and the envelope gene (1,425 bp) was completely sequenced by the dideoxinucleotide terminator method for 3 strains (5) by using the same set of oligonucleotides as for the RT-PCR amplification.

Phylogenetic analysis was performed by using the neighbor-joining method (6) and homologous sequences of different DENV-4 strains isolated in Central and South America (7,8). The Asian genotype II strains (Thailand 1978-U18441 and Thailand 1985-AY780644) were used as outgroups to give confidence to phylogenetic groupings. Phylogenetically, the DENV-4 strains grouped in genotype I and clustered with Venezuelan strains isolated from 2004 through 2007 (Brazil/Venezuela group) and were distantly related to strains isolated in Venezuela from 1998 through 2000. This result indicates that the current DENV-4 strains isolated in Roraima State were reintroduced to Brazil through Venezuela, where DENV-4 has circulated since the 1980s (1). This result also excludes the possibility that Asian genotypes previously circulated in Brazil. The DENV-4 strains isolated from patients in Roraima State in 2010 were genetically distinct from DENV-4 strains isolated in the 1980s (Figure, panel B).

During the 2010 outbreak, cocirculation of DENV-1 and DENV-2 in addition to DENV-4 was demonstrated by virus isolation (68 strains) and RT-PCR amplification (genome detection in 39 strains). The municipality of Boa Vista, which has ≈266,901 inhabitants, reported 5,243 dengue cases (3,936 dengue fever, 259 severe dengue or dengue hemorrhagic fever) in 2010 (epidemiologic week 37), many of them diagnosed by serologic testing (9). These numbers represent an increase of 154% over the 2,066 cases reported in 2009 (10).

DENV-4 had been introduced to Brazil through Boa Vista before the reemergence reported here; in 1982, DENV-1 and DENV-4 were described in Brazil, and a serologic survey estimated 11,000 dengue infections (1). After that outbreak, DENV-4 was eradicated and not again detected until the episode reported here. To monitor the circulation of DENV-4 in Roraima and other Brazilian states through the present date (epidemiologic week 48), strong serologic and virologic surveillance have been conducted; cases of this serotype have not been recognized outside Roraima State. As a preventive measure in Boa Vista, a total of 10,358 dwellings were visited and 18,305 larval *Aedes aegypti* mosquito foci were eliminated by spraying of mosquito adulticide. Nonetheless, as summer approaches in Brazil, a heavy rainy season is expected, and DENV-4 is a candidate to become a serious threat in the country. Therefore, the Ministry of Health has prepared a plan to investigate DENV-4 circulation; it calls for early detection of disease and adoption of control measures to avoid or minimize spread of this dengue serotype throughout the country.
